# Proteomics of Vitreous Humor Reveals PPARA, RXR, and LXR Are Possible Upstream Regulators of Proliferative Diabetic Retinopathy

**DOI:** 10.3389/fmed.2021.724695

**Published:** 2021-08-17

**Authors:** Siyan Li, Enzhong Jin, Xuan Shi, Yi Cai, Hui Zhang, Mingwei Zhao

**Affiliations:** ^1^Department of Ophthalmology, Peking University People's Hospital, Eye Diseases and Optometry Institute, Beijing Key Laboratory of Diagnosis and Therapy of Retinal and Choroid Diseases, College of Optometry, Peking University Health Science Center, Beijing, China; ^2^Department of Ophthalmology, Beijing Jingmei Group General Hospital, Beijing, China

**Keywords:** proliferative diabetic retinopathy, proteomics, vitreous humor, transcription factors, mass spectrometry

## Abstract

**Purpose:** To investigate the key regulators of the disease by comparing the abundance of vitreous proteins between the patients with proliferative diabetic retinopathy (PDR) and the controls with idiopathic epiretinal membrane (iERM).

**Methods:** Vitreous humor (VH) samples were derived from patients with PDR or iERM through the pars plana vitrectomy. The VH proteins were identified by liquid chromatography tandem mass spectrometry (LC-MS/MS) analysis. MaxQuant software and Metascape were applied to explore the enrichment of differentially expressed proteins in biological processes, cellular components, and molecular functions. Enrichr online tool and Gene Set Enrichment Analysis (GSEA) were performed to detect upstream transcriptional regulators of the highly expressed proteins.

**Results:** The present study collected 8 vitreous humor samples from 5 PDR eyes and 3 iERM eyes and identified 88 highly expressed proteins in PDR patients. We validated our highly expressed proteome was able to distinguish the PDR patients from the non-PDR patients by using the VH proteomics data from a previous study. The majority of highly expressed proteins were involved in complement and coagulation cascades, regulating exocytosis, and hemostasis. Using the Gene Set Enrichment Analysis (GSEA), we identified that transcription factors (TFs) PPAR-α, RXR, LXR regulate these proteins.

**Conclusions:** In this study, we identified a highly expressed proteome in VH of PDR patients. The role of the complement and coagulation system, regulating exocytosis, and hemostasis has been of great significance to PDR. Nuclear receptors PPARA, RXR, LXR were possible upstream regulators of disease progression and required further study.

## Introduction

Diabetic retinopathy (DR) has become the most prevalent cause of blindness among adults aged 20–74 and the major complication of patients with either type of diabetes (1). In the early, non-proliferative stage of the disease, DR begins with abnormal microvascular changes, which are characterized by microaneurysms, increased vascular permeability, capillary closures. With increasing duration, these microangiopathies can lead to neovascularization, indicates a proliferative stage developed. Proliferative diabetic retinopathy (PDR) is characterized by retinal neovascularization due to retinal ischemia. The overgrowth of the neovascular tufts toward the vitreous leading vitreous hemorrhage and fibrovascular membranes (FVMs) formation. In the severe stage of PDR, the FVMs can cause tractional retinal detachment and result in devastating vision impairment.

The progressive course of DR is often irreversible. Laser photocoagulation, intravitreal anti-vascular endothelial growth factor (VEGF) agents, and vitreoretinal surgery are classic treatments of the disease (2). Even if many patients have received proper treatment, it cannot deter the advancement of the disease. New therapeutic strategies are being explored in numerous ongoing trials, but most target the advanced stages of the disease. The complexity of pathophysiological mechanisms and molecular events contributing to DR creates obstacles in finding effective intervention in the very early stage.

The vitreous humor (VH) contains a variety of soluble proteins and has a close relationship with the progress of DR. Besides, the VH is the first site in the eye where anti-VEGF agents exert their curative effects. Many studies have demonstrated that label-free quantitative proteomics analysis is capable of detecting proteins in the VH and provided quantitatively mapped proteome changes of the VH in the DR patients (3). However, these data have not been fully used for further analysis and research to understand the complexity of pathophysiological mechanisms and molecular events contributing to the disease. In the present study, we firstly performed label-free quantitative proteomics analysis to identify the differentially expressed proteins between patients with PDR and patients with idiopathic epiretinal membrane (iERM). We determined 88 highly expressed proteins in PDR and found they can also distinguish PDR patients from non-PDR by using the previous VH protein profiles. Then we analyzed the upstream regulators of these proteins by combining our data with previous proteomics data. Using the Gene Set Enrichment Analysis (GSEA), we identified that transcription factors (TFs) including PPAR-α, RXR and LXR regulate these proteins.

## Materials and Methods

This was a cross-sectional, observational study of consecutive PDR patients who underwent surgical treatment at the Department of Ophthalmology, Peking University People's Hospital, Beijing, China, between March 2019 and December 2019. Patients in the control group underwent vitreoretinal surgery due to iERM. This study was approved by the Clinic Institutional Review Board of Peking University People's Hospital and complied with the Declaration of Helsinki. Written informed consent was obtained from all patients before enrollment in the present study. All patients underwent comprehensive preoperative eye examinations by the recruiting surgeons. Clinical data, including the medical history and treatment of diabetes mellitus, were collected. Each patient underwent eye examinations including visual acuity, intraocular pressure, axial length, slit-lamp biomicroscopy, dilated funduscopic examination, and optical coherence tomography before and after surgery.

VH samples (up to 200 μL) were collected before conventional three-port pars plana vitrectomy without artificial humor infusion. A 25-gauge trocar was introduced into the inferior temporal sclera, and a closed infusion tube was inserted. The stopcock of the vitrector aspiration line was opened, and a 5 mL sterile syringe was attached. By active cutting combined with syringe suction, 100–200 μL vitreous was aspirated into the syringe, the aspiration line was closed, and the infusion was initiated to stabilize the intraocular pressure. The samples were transferred into sterile 1.5 mL microcentrifuge tubes, snap-frozen, and stored at −80°C until further analysis.

VH samples were centrifuged at 20,000 g for 15 min at 48°C to remove cells or cell debris. Nanodrop determined the concentration of protein. For liquid chromatography tandem mass spectrometry (LC-MS/MS) analysis, samples were separated by a 120 min gradient elution at a flow rate of 0.300 μL/min with the Thermo Ultimate 3000 nano-UPLC system which was directly interfaced with the Thermo Fusion LUMOS mass spectrometer. The analytical column was an Acclaim PepMap RSLC column (75 μm ID, 250 mm length, C18). Mobile phase A consisted of 0.1% formic acid, and mobile phase B consisted of 100% acetonitrile and 0.1% formic acid. The Fusion LUMOS mass spectrometer was operated in the data-dependent acquisition mode using Xcalibur 4.1.50 software, and there is a single full-scan mass spectrum in the Orbitrap (375–1,500 m/z, 60,000 resolution) followed by data-dependent MS/MS scans. The MS/MS spectra from each LC-MS/MS run were searched against the selected database using the software Proteome Discovery (version 2.2).

Label-free quantification was performed using MaxQuant software (version 1.5.3.30), and the iFOT (defined as iBAQ/iBAQ_total) values were used to quantify protein expression. The cutoff of the false discovery rate for peptide and protein identification was set to 0.05. Gene ontogeny (GO) analysis was applied to explore the possible biological functions of the differentially expressed proteins via Metascape (4); *p*-values of Fisher's exact test were calculated to measure the significance of enriched ontology terms and pathways.

TFs and target genes of each TF were extracted from ChIP-seq data in the ChEA database (5). TFs that regulate highly expressed proteins were enriched using the Enrichr online tool (6). The predicted regulating TFs were validated in an external validation set (7). Gene Set Enrichment Analysis (GSEA) was applied to quantify the activity of each candidate TF in different samples using the GSEApy package (https://github.com/zqfang/GSEApy).

## Results

### Patient Information

A total of eight patients (8 eyes) was recruited in our study, including 5 PDR eyes and 3 non-diabetic eyes (control). Three eyes underwent vitreoretinal surgery due to tractional retinal detachment, and 2 eyes due to persistent vitreous hemorrhage. Three non-diabetic eyes underwent surgery due to iERM. The two groups were well-balanced for demographics. The VH protein concentrations were not significantly different among the two groups. The characteristics of the PDR and control patients were summarized in Table 1.

**Table 1:** Data for patients with proliferative diabetic retinopathy or idiopathic epiretinal membrane.

**Characteristics**	**Idiopathic epiretinal membrane group (*n* = 3)**	**Proliferative diabetic retinopathy group (*n* = 5)**
Age (year)	63 ± 10.61	60.4 ± 9.71
Sex, male/female	2/1	3/2
Diabetes duration (year)	–	14.1 ± 10.70
Macular edema	–	2
Vitreous hemorrhage	–	3
Traction membrane	–	3
Proliferative membrane	–	3
Tractional retinal detachment	–	2
Retinal laser frequency	–	1.6 ± 1.36
Insulin	–	4
Hypoglycemic drugs	–	2

**Figure 1:**
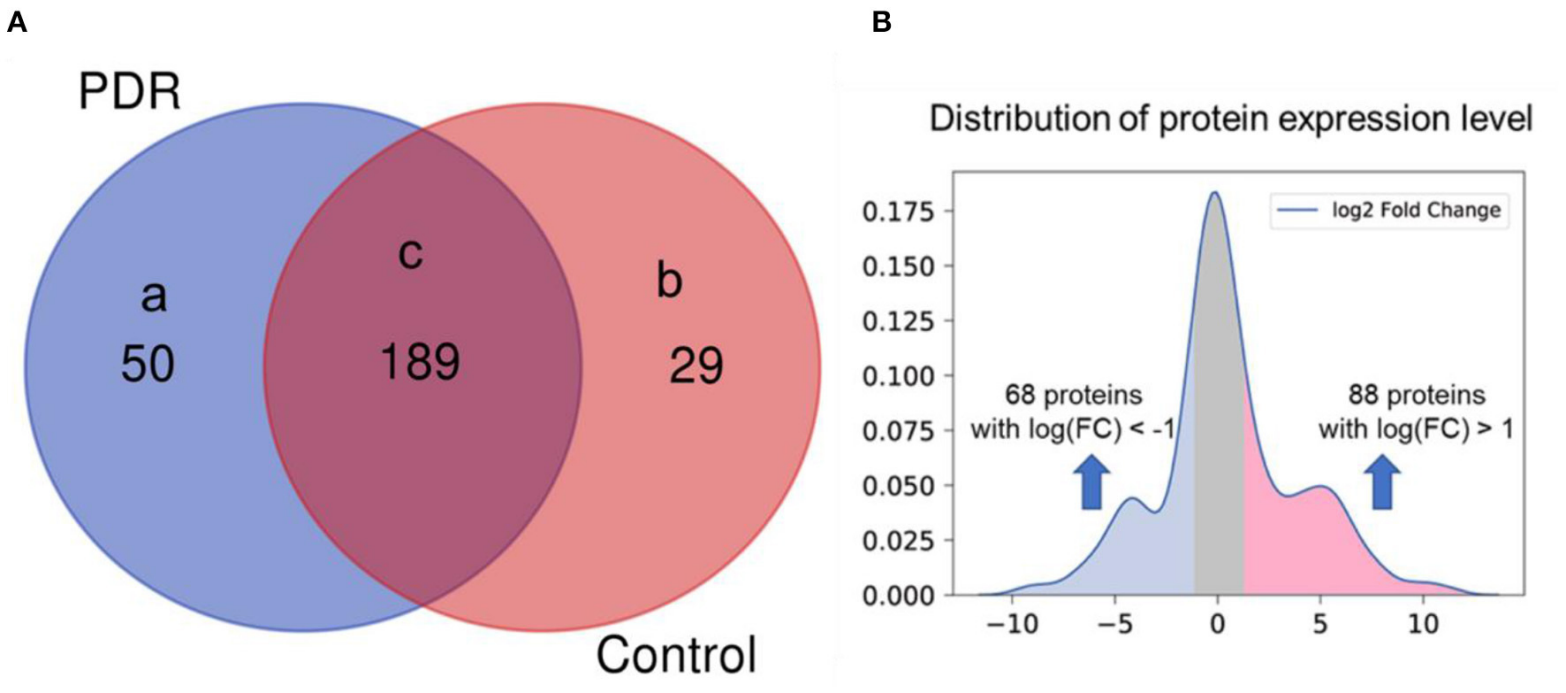
Differentially expressed proteins between PDR patients and control. **(A)** Proteins detected in (a) only PDR group (*n* = 5), (b) only control group (*n* = 3), (c) both groups. **(B)** Distribution of protein expression level in PDR patients (log2 fold change of expression level in PDR to control, x-axis) and significance (*P*-value, y-axis).

After proteome profiling, differentially expressed proteins were identified between PDR and control group as shown in Figure 1 proteomic analysis identified 239 and 218 intravitreal proteins by LC/MS-MS in PDR and control group. Among these proteins, 50 proteins were detected only in the PDR group, and 29 proteins were detected only in the control group. Among the total 268 proteins, 189 proteins were detected in both groups, 88 proteins were significantly highly expressed in PDR patients, and 68 proteins were significantly low expressed in PDR patients. We defined those 88 highly expressed proteins as the highly expressed proteome.

**Figure 2:**
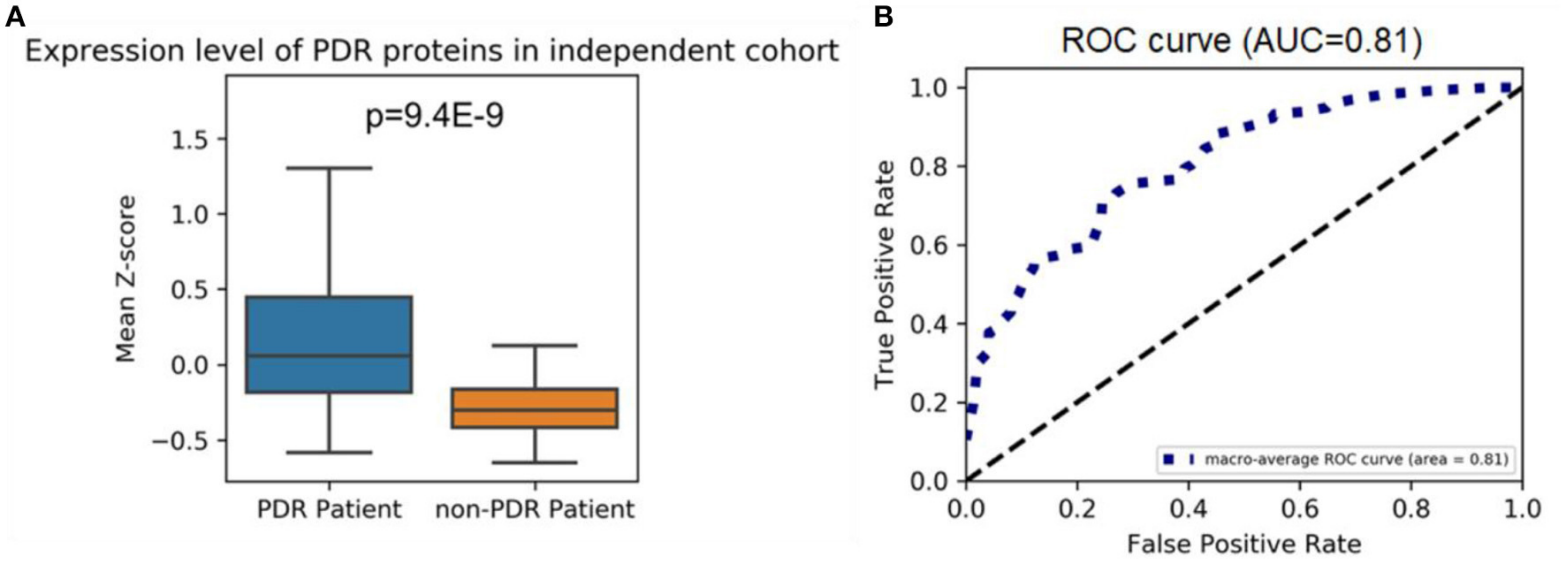
**(A)** Highly expressed proteome levels in PDR and non-PDR patients from an independent cohort; the mean Z-scores of highly expressed proteins in each patient from two groups were shown. A one-tailed Mann–Whitney *U*-test was applied to calculate the *p*-value. **(B)** ROC curve for the highly expressed proteomes at separating PDR patients from non-PDR patients. ROC curves are plots of the true positive rate (vertical axis) against the false positive rate (horizontal axis) for the different possible cutoff points of a diagnostic test.

We used the VH proteomics data from a previous quantitative proteomics analysis study as an independent validation set to validate the highly expressed proteome is specific in PDR patients (7). The highly expressed proteome in our cohort remained highly expressed in PDR patients in the independent cohort (*P* = 9.4E-9, Figure 2A). Figure 2B shows the ROC curve and AUC which examine the performance of the mean Z-score of the highly expressed proteome to separate PDR patients from the non-PDR patients.

### Gene Ontology Enrichment Analysis

**Figure 3:**
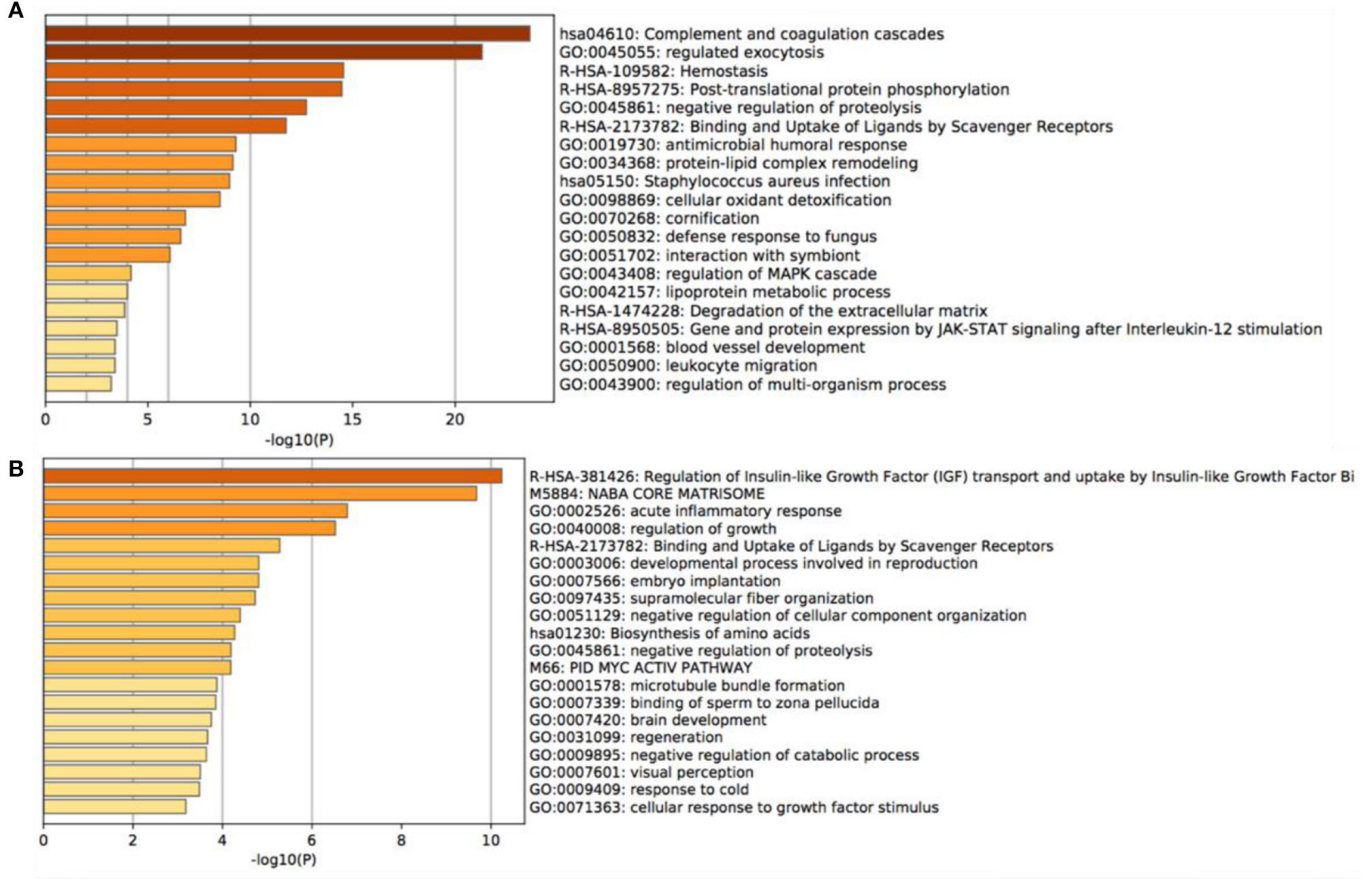
Gene Ontology enrichment analysis of the main altered proteins. **(A)** the most notable 20 GO annotations of the increased proteins, **(B)** the most notable 20 GO annotations of the decreased proteins.

Gene enrichment analysis was performed to identify the enrichment of differentially expressed proteins in biological processes, cellular components, and molecular functions. The results demonstrated that the majority of highly expressed proteins were involved in complement and coagulation cascades, regulating exocytosis, and hemostasis (Figure 3A). Conversely, the low expressed proteins were mainly associated with the regulation of insulin-like growth factor transport and uptake by insulin-like growth factor Bi (Figure 3B).

### Enrichr Analysis and TFs Identification

**Figure 4:**
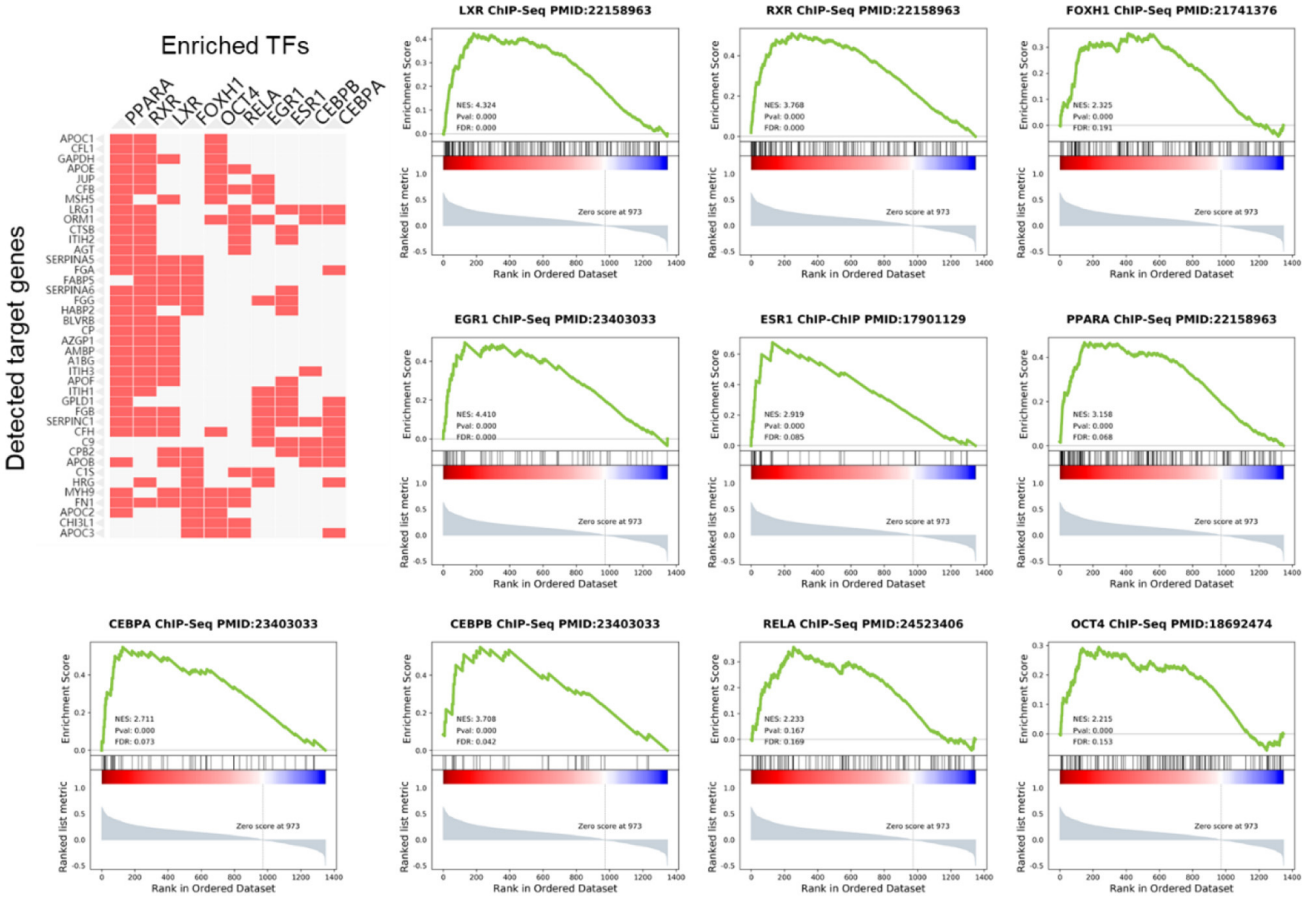
Gene Set Enrichment Analysis (GSEA) of TFs in PDR patients. The cluster gram shows highly expressed proteins and top 10 upstream TFs inferred from ChIP-seq data.

We analyzed the upstream TFs which promote the expression of these PDR-related proteins in the ChEA database. As the result, most of the highly expressed proteins were regulated by peroxisome proliferative-activated receptor alpha (PPAR-α), retinoid-X receptor (RXR), Liver X receptor (LXR), forkhead activin signal transducer 1 (FOXH1), octamer-binding transcription factor 4 (OCT4), RELA/transcription factor p65, early growth response 1 (EGR1), estrogen receptor 1 (ESR1), enhancer-binding protein α (CEBPA), and enhancer-binding protein β (CEBPB) (Figure 4).

**Figure 5:**
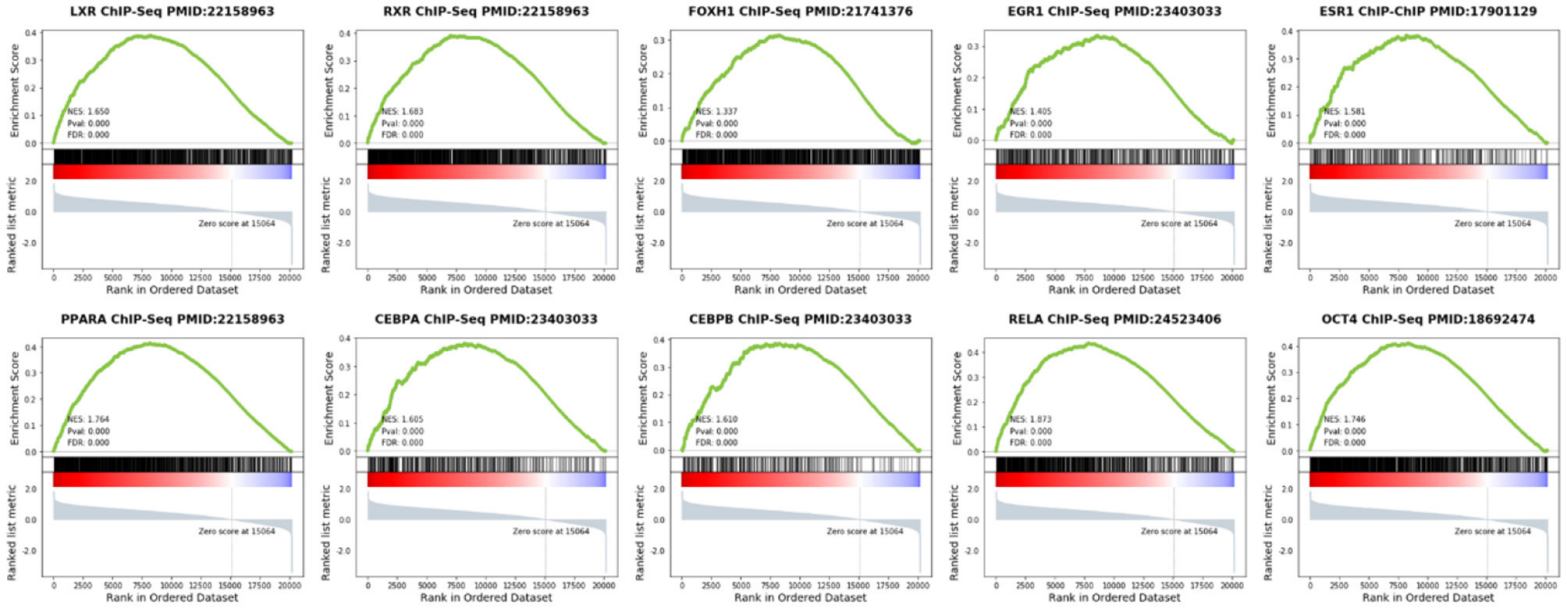
Gene Set Enrichment Analysis (GSEA) of TFs in vascular endothelial cells obtained from FVMs compared with control retinal endothelial cells.

Using GSEA in the previous PDR/non-PDR proteome study (7), we identified target genes of TFs including PPARA, RXR, LXR, FOXH1, OCT4, RELA, EGR1, ESR1, CEBPA, and CEBPB were significantly up-regulated in the proteome data of PDR patients in the independent cohort. We reanalyzed the transcriptome of vascular endothelial cells obtained from FVMs in a previous study (8), and consistently, the results demonstrated that gene targets of these TFs were also up-regulated in the PDR related vascular endothelial cells (Figure 5).

## Discussion

We performed label-free quantitative proteomics analysis to compare the vitreous humor (VH) of PDR patients and iERM patients, and identified proteins explicitly expressed within the PDR patients. A few proteomics studies focused on the VH in DR, but most of them did not compare PDR patients and non-DR patients. Li et al. conducted the proteomic analysis on VH from PDR patients and idiopathic macular hole patients and identified 52 proteins over-expressed in PDR patients out of 610 proteins (3). In this study, though the number of proteins we demonstrated was less than them, more highly expressed proteins (88 proteins) were identified compared with iERM. Since the collection of an ordinary person's VH is unethical, we suggest iERM patients are more suitable as controls. The highly expressed collection of proteins can also properly separate PDR patients from non-PDR patients in the most extensive VH proteomics analysis by far (7), suggesting amounts of proteins in the vitreous humor of diabetic retinopathy patients might indicate the progress of proliferation. It can be used to understand the comprehensive molecular pathogenesis of PDR and help with individualized treatment.

We performed Gene enrichment analysis to identify the enrichment of differentially expressed proteins in biological processes, cellular components, and molecular functions. We noticed that the proteins up-regulated in the PDR samples most significantly were attributed to complement and coagulation cascades. The role of the complement and coagulation system in angiogenesis has become of prime importance. Previous studies indicated that the complement components were present in the early stages of the DR (9–11). Indeed, the proteomics profile of our enriched results indicated the abundance of cascade components increased dramatically in the proliferative stage. Our results suggest that the role of the complement system might be an important mediator of DR pathogenesis. Previous research demonstrated prolonged exposure to elevated glucose alters the exocytosis in retinal neurons, which contributes to the pathogenesis of DR (12). Notably, we demonstrated that the 27 highly expressed proteins (including CAT, CHI3L1, FABP, CTSB, etc.) were functionally involved in the regulation of exocytosis. These results suggest that exocytosis is the major pathologic event in PDR, and future researches need to focus on the exocytosis mechanism in the development of PDR.

Presently, there are already some proteomics researches on PDR, however, these data have not been fully used for further analysis and research. Since we found the highly expressed proteome in our cohort remained highly expressed in PDR patients in the largest-scale cohort (7), we sought to determine the upstream regulator of these proteins. Therefore, enrichment analysis was performed by the Enrichr online tool, and we identified the top 10 TFs including PPARA, RXR, LXR, FOXH1, OCT4, RELA, EGR1, ESR1, CEBPA, and CEBPB regulated these highly expressed proteins. Then, GSEA analysis was performed to elucidate the target genes regulated by these identified TFs in PDR patients. Our results showed that the expression of these genes was elevated in PDR. We demonstrated that the target genes of the top 10 TFs were overexpressed in FVMs. Therefore, our results highlighted these TFs play an important role in the pathogenesis of PDR. The highly expressed proteins in VH might be released from FVMs in PDR. The present study is the first to utilize bioinformatics tools based on previous proteomics data of VH in PDR.

Among those transcription factors, PPARA, RXR, and LXR are nuclear receptors deserving more concern. PPARA, also known as PPARα, is one of the three subtypes of peroxisome proliferator-activated receptors (PPARs). It plays a crucial role in the regulation of ketogenesis, lipid transport, lipogenesis, cholesterol metabolism, fatty acid transport, and oxidation (13). For PPARs to induce gene expression, they must also interact with their co-activator, the retinoid-X receptor (RXR) (14). Previous studies related to decreased PPARA expression in diabetic retinas contributed to retinal inflammation and neovascularization in DR (15). Cells treated with RXR agonists were demonstrated to prevent the effect of high glucose (16). Besides, activation of LXR was reported to prevent inflammation and the formation of diabetes-induced acellular capillaries (17). All these studies revealed the upregulation of PPARA/RXR/LXR contributed to DR progression delay by playing a protective role in the retina. However, in the current study, we found that highly expressed proteins were regulated by PPARA/RXR/LXR, suggesting that these TFs might be activated in the stage of PDR. Compared with a study of saliva samples collected from DR patients, the differentially expressed proteins also indicated increased LXR/RXR activation (13). Our study provided the evidence for the first time that PPARA/RXR/LXR was activation in PDR. There should be careful consideration of the use of PPARA/RXR/LXR agonists recommended by previous studies. The comprehensive mechanism, as well as for drug instructions, needs to be explored by future studies.

The primary limitations of this study were the scale of the enrolled patients and the number of proteins we identified, which limited the available pool of data to analyze. Non-DR patients with iERM were enrolled as controls, but they were not disease-free status. Some iEMR-related proteins in the VH might have affected the differentiation from the PDR group.

## Conclusions

In this study, we identified a highly expressed proteome in VH of PDR patients. The role of the complement and coagulation system, regulating exocytosis, and hemostasis has been of great significance to PDR. Nuclear receptors PPARA, RXR, LXR were possible upstream regulators of disease progression and required further study.

## Data Availability Statement

The data presented in the study are deposited in the ProteomeXchange repository with accession number PXD027556, and the iProX repository with accession number IPX0003325000.

## Ethics Statement

The studies involving human participants were reviewed and approved by The Ethics Committee of Peking University People's Hospital. The patients/participants provided their written informed consent to participate in this study.

## Author Contributions

SL and MZ conceived the study and wrote the paper with input from all authors. SL and EJ designed the research and performed the research. SL, EJ, and XS collected the samples and data. SL, XS, and HZ analyzed the data. All authors contributed to the article and approved the submitted version.

## Conflict of Interest

The authors declare that the research was conducted in the absence of any commercial or financial relationships that could be construed as a potential conflict of interest.

## Publisher's Note

All claims expressed in this article are solely those of the authors and do not necessarily represent those of their affiliated organizations, or those of the publisher, the editors and the reviewers. Any product that may be evaluated in this article, or claim that may be made by its manufacturer, is not guaranteed or endorsed by the publisher.

## References

[1] Fong DS Aiello L Gardner TW King GL Blankenship G Cavallerano JD. Retinopathy in Diabetes. Diabetes Care. (2004) 27(suppl 1):s84–s7. 10.2337/diacare.27.2007.S84 14693935

[2] Mohamed Q Gillies MC Wong TY. Management of diabetic retinopathy: a systematic review. JAMA. (2007) 298:902–16. 10.1001/jama.298.8.902 17712074

[3] Li J Lu Q Lu P. Quantitative proteomics analysis of vitreous body from type 2 diabetic patients with proliferative diabetic retinopathy. BMC Ophthalmol. (2018) 18:151. 10.1186/s12886-018-0821-3 29940965 PMC6020172

[4] Zhou Y Zhou B Pache L Chang M Khodabakhshi AH Tanaseichuk O. Metascape provides a biologist-oriented resource for the analysis of systems-level datasets. Nat Commun. (2019) 10:1523. 10.1038/s41467-019-09234-6 30944313 PMC6447622

[5] Lachmann A Xu H Krishnan J Berger SI Mazloom AR Ma'ayan A. ChEA: transcription factor regulation inferred from integrating genome-wide ChIP-X experiments. Bioinformatics. (2010) 26:2438–44. 10.1093/bioinformatics/btq466 20709693 PMC2944209

[6] Kuleshov MV Jones MR Rouillard AD Fernandez NF Duan Q Wang Z. Enrichr: a comprehensive gene set enrichment analysis web server 2016 update. Nucleic Acids Res. (2016) 44:W90–7. 10.1093/nar/gkw377 27141961 PMC4987924

[7] Loukovaara S Nurkkala H Tamene F Gucciardo E Liu X Repo P. Quantitative proteomics analysis of vitreous humor from diabetic retinopathy patients. J Proteome Res. (2015) 14:5131–43. 10.1021/acs.jproteome.5b00900 26490944

[8] Lam JD Oh DJ Wong LL Amarnani D Park-Windhol C Sanchez AV. Identification of RUNX1 as a mediator of aberrant retinal angiogenesis. Diabetes. (2017) 66:1950–6. 10.2337/db16-1035 28400392 PMC5482092

[9] Yanai R Thanos A Connor KM. Complement involvement in neovascular ocular diseases. Adv Exp Med Biol. (2012) 946:161–83. 10.1007/978-1-4614-0106-3_10 21948368

[10] Huang C Fisher KP Hammer SS Busik JV. Extracellular vesicle-induced classical complement activation leads to retinal endothelial cell damage via MAC deposition. Int J Mol Sci. (2020) 21:1693. 10.3390/ijms21051693 32121610 PMC7084203

[11] Shahulhameed S Vishwakarma S Chhablani J Tyagi M Pappuru RR Jakati S. A systematic investigation on complement pathway activation in diabetic retinopathy. Front Immunol. (2020) 11:154. 10.3389/fimmu.2020.00154 32117292 PMC7026189

[12] Baptista FI Castilho ÁF Gaspar JM Liberal JT Aveleira CA Ambrósio AF. Long-term exposure to high glucose increases the content of several exocytotic proteins and of vesicular GABA transporter in cultured retinal neural cells. Neurosci Lett. (2015) 602:56–61. 10.1016/j.neulet.2015.06.044 26141610

[13] Chee CS Chang KM Loke MF Angela Loo VP Subrayan V. Association of potential salivary biomarkers with diabetic retinopathy and its severity in type-2 diabetes mellitus: a proteomic analysis by mass spectrometry. PeerJ. (2016) 4:e2022. 10.7717/peerj.2022 27280065 PMC4893325

[14] Treacy MP Hurst TP. The case for intraocular delivery of PPAR agonists in the treatment of diabetic retinopathy. BMC Ophthalmol. (2012) 12:46. 10.1186/1471-2415-12-46 22937835 PMC3532122

[15] Hu Y Chen Y Ding L He X Takahashi Y Gao Y. Pathogenic role of diabetes-induced PPAR-α down-regulation in microvascular dysfunction. Proc Natl Acad Sci USA. (2013) 110:15401–6. 10.1073/pnas.1307211110 24003152 PMC3780907

[16] Chai D Wang B Shen L Pu J Zhang X-k He B. RXR agonists inhibit high-glucose-induced oxidative stress by repressing PKC activity in human endothelial cells. Free Radic Biol Med. (2008) 44:1334–47. 10.1016/j.freeradbiomed.2007.12.022 18206668

[17] Hammer SS Beli E Kady N Wang Q Wood K Lydic TA. The mechanism of diabetic retinopathy pathogenesis unifying key lipid regulators, sirtuin 1 and liver X receptor. EBioMedicine. (2017) 22:181–90. 10.1016/j.ebiom.2017.07.008 28774737 PMC5552206

